# Characterization of a core fragment of the rhesus monkey TRIM5α protein

**DOI:** 10.1186/1471-2091-12-1

**Published:** 2011-01-04

**Authors:** Alak K Kar, Youdong Mao, Gregory Bird, Loren Walensky, Joseph Sodroski

**Affiliations:** 1 Department of Cancer Immunology and AIDS, Dana-Farber Cancer Institute, Harvard Medical School, Boston, MA, USA 02115; 2 Department of Pathology, Division of AIDS, Harvard Medical School, Boston, MA, USA 02115; 3 Department of Pediatric Oncology, Dana-Farber Cancer Institute, Harvard Medical School, Boston, MA, USA 02115; 4 Department of Immunology and Infectious Disease, Harvard School of Public Health, Boston, MA, USA 02115

## Abstract

**Background:**

Like all tripartite motif (TRIM) proteins, the retroviral restriction factor TRIM5α consists of RING, B-box 2 and coiled-coil domains, with a C-terminal B30.2(SPRY) domain. Although structures have been determined for some individual TRIM domains, the structure of an intact TRIM protein is unknown.

**Results:**

Here, we express and characterize a protease-resistant 29-kD core fragment containing the B-box 2, coiled coil and adjacent linker (L2) region of TRIM5α. This BCCL2 protein formed dimers and higher-order oligomers in solution. Approximately 40% of the BCCL2 secondary structure consisted of alpha helices. Partial loss of alpha-helical content and dissociation of dimers occurred at 42°C, with the residual alpha helices remaining stable up to 80°C.

**Conclusions:**

These results indicate that the B-box 2, coiled-coil and linker 2 regions of TRIM5α form a core dimerization motif that exhibits a high level of alpha-helical content.

## Background

Soon after entry into the cells of certain mammalian species, some retroviruses encounter blocks mediated by the restriction factor TRIM5α. For example, human immunodeficiency virus (HIV-1), the causative agent of acquired immunodeficiency syndrome (AIDS), can infect human and chimpanzee cells, but is blocked in cells of Old World monkeys [1-5]. In contrast, simian immunodeficiency virus (SIVmac) infects Old World monkey cells but is restricted in the cells of most New World monkeys [3,6]. TRIM5α, a member of the tripartite motif (TRIM) family of proteins, mediates these early infection blocks [7-12]. Differences in TRIM5α proteins among species account for the distinct patterns of retroviral restriction observed in mammalian lineages [5,7-12]. TRIM5α has been shown to bind and promote the premature uncoating of incoming retroviral capsids [13-19].

Like all TRIM proteins, TRIM5α contains RING, B-box 2 and coiled coil domains [20,21]. The α isoform of TRIM5, which is the only TRIM5 isoform that exhibits antiretroviral activity [7], also has a carboxy-terminal B30.2(SPRY) domain [20,21]. Mutagenic studies have shed light on the contribution of the TRIM5α domains to antiretroviral activity. The RING domain, which exhibits E3 ubiquitin ligase activity, contributes to anti-HIV-1 potency, but is not absolutely required for a modest level of restriction ability [7,13,22-24]. The B-box 2 domain is essential for higher-order associations among TRIM5α dimers and contributes to the cooperative binding of TRIM5α to the retroviral capsid [14,16,25,26]. The coiled coil and adjacent linker (L2) region are sufficient for dimerization of TRIM5α, which results in a higher avidity of TRIM5α-capsid binding [27-30]. The direct contact between TRIM5α and the retroviral capsid is thought to be mediated by the B30.2(SPRY) domain. Surface-exposed loops within the TRIM5α B30.2(SPRY) domain exhibit pronounced variability and have been subject to positive selection during mammalian evolution [31-36]. Amino acid changes in these B30.2(SPRY) variable loops or in the viral capsid protein can alter capsid recognition and the specificity of retrovirus restriction [15,23,33,37-47].

Structural information on TRIM proteins is still limited. There are no published structures of a complete TRIM protein, even though some TRIM proteins (e.g., TRIM19 (promyelocytic leukemia (PML) protein), TRIM20 (pyrin), TRIM18 (MID1), TRIM21 (Rho/SSA)) and TRIM37 (MUL)) have been studied extensively due to their association with human diseases [48-54]. Poor expression levels and tendencies to aggregate into nuclear or cytoplasmic bodies have hampered efforts to obtain complete TRIM protein structures [20,21]. Nonetheless, structures of individual TRIM domains have been solved. The RING and B-box 2 structures of TRIM5 and other TRIM proteins have been solved by NMR [16,55-59]. Structures of the B30.2(SPRY) domains of TRIM21 as well as of several proteins not in the TRIM family have been obtained by x-ray crystallography [60-64]. No structural information on the coiled-coil domain of any TRIM protein is available. Such information would be very valuable, as the coiled coil presumably resides near the dimeric axis and provides a central scaffold for the positioning of the other TRIM domains. The TRIM coiled coil is predicted to be alpha helical [65-67], but lacks easily identifiable heptad repeat motifs that characterize some of the classical coiled coils for which structural information is available.

Recently, we have devised approaches that allow expression of sufficient quantities of TRIM5α variants in baculovirus-infected cells for purification and biochemical characterization [28,29]. Substitution of the TRIM21 RING domain for that of rhesus monkey TRIM5α resulted in a protein (TRIM5-21R) that was efficiently expressed and was purified. TRIM5-21R is a dimer and binds HIV-1 capsid-nucleocapsid (CA-NC) complexes assembled in vitro[28,29]. Binding of TRIM5-21R to HIV-1 CA-NC complexes depends upon the B30.2(SPRY) domain v1 variable region. Protease-resistant fragments of TRIM5-21R were characterized [28]. Here we describe the expression in E. coli, purification and characterization of one such fragment containing the B-box, coiled coil and linker 2 (L2) region of rhesus monkey TRIM5α.

## Methods

### Plasmid construction and production of recombinant BCCL2 protein

The recombinant bacterial construct expressing BCCL2 was prepared using the Champion pET Gateway vector with a kanamycin cassette(Invitrogen). Gateway recombination resulted in a gene that encodes an N-terminal hexa-histidine tag, a spacer, and the BCCL2 protein. The predicted N-terminus of the resulting BCCL2 protein is HHHHHHGSGLVPRGSASMSDSEVNQEAKPEVKLSPEEGQK.... The recombinant vector was transformed into BL21(DE3)pLysS competent cells (Invitrogen). Five single colonies were picked and examined for expression. The colony exhibiting the highest level of BCCL2 expression was incubated in Luria Broth (LB) at 37°C till the OD reached 0.8, at which time isopropyl-thiogalactoside (IPTG) was added to a final concentration of 1 mM; incubation was continued overnight at 18°C. The cDNAs expressing the wild-type BCCL2 protein and B-box 2 mutants were cloned into pcDNA/V5-GW/D-TOPO (Invitrogen) for expression in mammalian cells.

### Expression and purification of the BCCL2 proteins

Single bacterial colonies were expanded and induced as described above. The cells were harvested by centrifugation at 4,000 × *g *for 10 minutes, rinsed in phosphate-buffered saline, and resuspended at 2.5 × 10^7 ^cells/ml in Bacterial Protein Extraction Reagent (BPER) (Pierce Biotechnology) in the presence of 1 mM dithiothreitol (DTT) and a cocktail of protease inhibitors comprising 10 mM (amidinophenyl)-methanesulfonyl fluoride (APMSF), 10 mM E-64, 10 mM leupeptin, and 1 mM pepstatin A (final concentrations). A sample was taken for analysis, and then the cell debris and nuclei were removed by centrifugation at 4,000 × *g *for 10 minutes. The resulting cell supernatant was mixed with Ni^+2^-nitrilotriacetic acid (NTA) superflow resin (Qiagen) according to the manufacturer's protocol. Protein was eluted from the resin with 300 mM imidazole. The eluted protein fraction was concentrated and was loaded on a Superdex 200 HR 10/30 size-exclusion chromatography (SEC) column (GE Healthcare). The column was equilibrated against PBS. Preparative SEC was run at 0.2 ml/min at 4°C. A calibration curve for molecular size estimation was generated by loading gel-filtration markers (Biorad) comprising thyroglobulin (667 kD), bovine gamma-globulin (158 kD), chicken ovalbumin (44 kD), equine myoglobin (17 kD) and Vitamin B_12 _(1.35 kD) proteins onto this SEC column and eluting under similar conditions. The resulting fractions were analyzed by SDS-PAGE and those containing the BCCL2 protein were pooled and concentrated. For further purification, buffer was exchanged into 20 mM Tris-HCl/50 mM NaCl, pH 8 by using a PD-10 column (Pharmacia). Anion-exchange chromatography was subsequently performed on a 5-ml Q-Sepharose Hi-Trap column (GE Healthcare) equilibrated with 20 mM Tris-HCl/50 mM NaCl, pH 8.0. Bound proteins were eluted by using a NaCl gradient (50 to 750 mM NaCl in 20 mM Tris-HCl, pH 8.0). The BCCL2 protein was eluted with 400 mM NaCl, and fractions containing the protein were pooled, dialyzed and concentrated by ultrafiltration with Centricon-10 devices (Amicon). The cocktail of protease inhibitors (at the concentrations referred to above) was added to the samples throughout each stage of the purification scheme. The BCCL2 protein was at least 95% pure, as determined by a Coomassie-stained gel.

### Elicitation of polyclonal antibodies in rabbits

Polyclonal antibodies directed against the purified LLER protein were raised in rabbits by Open Biosystems (Huntsville, AL, USA). The rabbits were primed with 100 μg of protein in Complete Freund's Adjuvant and boosted twice with 50 μg protein in Incomplete Freund's Adjuvant.

### Western blotting

Aliquots were collected at each step of BCCL2 purification and the associated proteins were resolved by sodium dodecyl sulfate-polyacrylamide gel electrophoresis (SDS-PAGE). The proteins were transferred to poly(vinylidene fluoride) (PVDF) Immobilon filters (Millipore) by a semidry blotting apparatus. Polyclonal rabbit antibody directed against the purified BCCL2 protein was used as the primary antibody to detect the BCCL2 protein in Western blot experiments. Horseradish peroxidase (HRP)-conjugated goat anti-rabbit IgG was used as the secondary antibody. The Western blot was developed with the ECL chemiluminescence detection system (GE Healthcare) and the Kodak film processor system (Kodak).

### Analytical size-exclusion chromatography

A sample of the Ni^+2^-affinity-purified BCCL2 protein was fractionated by size exclusion on a Superdex 200 (10/30) column calibrated with molecular-weight markers. The column was equilibrated in PBS at a flow rate of 0.4 ml/min, and the absorbance of the eluted protein was recorded at 280 nm. The column was mounted on a high-performance liquid chromatography system (Varian Star). Fractions were collected every minute, and a sample from each fraction was analyzed by SDS-PAGE.

### Size-Exclusion Chromatography-Light Scattering (SEC-LS)

The molecular masses of full-length proteins were determined by Ewa Folta-Stogniew using SEC-LS in the HHMI Biopolymer Facility and W. M. Keck Foundation Biotechnology Resource Laboratory at Yale University. Briefly, a sample containing approximately 300 μg of LLER protein was filtered through a 0.22-μm Durapore membrane (Millipore) and applied to a Superose 6 HR 10/30 column coupled with an in-line Dawn EOS laser light-scattering apparatus (Wyatt Technology Corporation), refractometer (Wyatt Technology), and UV detector (Waters Corporation). The average molecular mass of the elution peak was calculated using ASTRA software.

### Mass spectrometry

Matrix-assisted laser desorption ionization-time of flight (MALDI-TOF) mass spectrometry of the LLER protein was performed at the Molecular Biology Core Facility at Dana-Farber Cancer Institute (Boston, MA), using a sinapinic acid matrix.

### Dynamic Light Scattering

Measurements were carried out using a temperature-controlled Zetasizer Nano-S (Malvern) at 20°C. A protein solution (0.5 mg/ml) was filtered through a 0.2-μm membrane into a 20°C pre-warmed light-scattering cuvette and measurements were carried out according to the manufacturer's instructions.

### Crosslinking of the purified BCCL2 protein

The purified LLER protein (1 mg/ml) was incubated with various concentrations (final concentrations of 0, 0.25, 0.5, 1 and 1.0 mM) of glutaraldehyde (Sigma) at room temperature for 8 minutes, after which 0.1 M Tris-HCl, pH 7.5 was added to quench the reaction. The crosslinked proteins were boiled in SDS-denaturing buffer and subjected to SDS-PAGE (4-12% acrylamide) and Coomassie Blue staining.

Transiently transfected 293 T cells expressing wild-type rhesus monkey BCCL2 and the corresponding B-box 2 mutants were washed in phosphate-buffered saline (PBS) and lysed in NP40 lysis buffer (0.5% Nonidet P40 (NP40), 1× protease inhibitor (complete EDTA-free, Roche Diagnostics) in PBS) for 45 minutes at 4°C. Lysates were centrifuged at 14,000 × *g *for 15 minutes at 4°C. Lysates were crosslinked with varying concentrations (up to 2 mM) of glutaraldehyde for 8 minutes at room temperature and centrifuged briefly in a table-top centrifuge. The reaction mix was quenched with 0.1 M Tris-HCl, pH 7.5, briefly centrifuged and analyzed by Western blotting as described above.

### Native gel electrophoresis

Electrophoresis was performed using the Novex Polyacrylamide Gel Electrophoresis System (Invitrogen). Protein samples were mixed with native gel running buffer (0.1 M Tris-HCI (pH 8.8), 20% glycerol, and 0.0025% bromophenol blue) to a final concentration of 0.5 mg/ml. Thirty microliters of each sample was loaded onto a 4-16% gradient polyacrylamide gel and run at 150 V in Tris-glycine buffer (0.192 M acetate and 0.25 M Tris at pH 8.3). After the dye front migrated to the bottom of the gel, the gel was developed using Coomassie Blue staining. The unstained NativeMark protein standard (Invitrogen) was used for markers.

### Electron Microscopy

For negative staining, the purified LLER protein was directly applied to carbon-coated grids after glow discharge. These were negatively stained with 1% (w/v) uranyl formate and observed in a Tecnai G2 Spirit BioTWIN (FEI Company) electron microscope operated at 100 kV. Micrographs were recorded at a magnification of 100,000 x and pictures taken under low-dose conditions.

For cryo-electron microscopic analysis, 3 μl of the protein solution was applied to a Quantifoil grid, blotted by filter paper for 2 seconds and immediately plunged into liquid ethane; the whole process was performed with an FEI Vitrobot. The prepared cryo-grids then were subjected to imaging in a Tecnai F20 field-emission gun electron microscope equipped with a Gatan CT3500 cryo-transfer holder that was cooled down to -180°C by liquid nitrogen. Micrographs were recorded in bright-field mode at a magnification of 150,000 × and at a defocus of ~3-5 μm by a 4 k × 4 k Gatan CCD camera, with a total dose no greater than 100 electrons per nm^2^.

### Circular dichroism (CD) spectroscopy

Samples for circular dichroism (CD) spectroscopy were buffer-exchanged into PBS. The CD spectrum was measured with an Aviv 410 CD spectrometer (Aviv Biomedical), using a quartz cuvette with a path length of 0.1 cm. Measurements were obtained in 0.5-nm intervals from 195 to 245 nm, a 1-nm bandwidth, and a 0.5-s measurement time at each wavelength. Instrument units were converted to mean residue molar ellipticity according to the formula: θ = millidegree/molar concentration/number of amino acids. The percentage of secondary structure was calculated using the following formula: % helicity = 100 × θ_222_(observed)/θ_222_(max), where θ_222_(max) = -40,000 × [1 - (2.5/number of amino acid residues)].

## Results

### Expression and purification of a core fragment of TRIM5α_rh_

**Figure 1:**
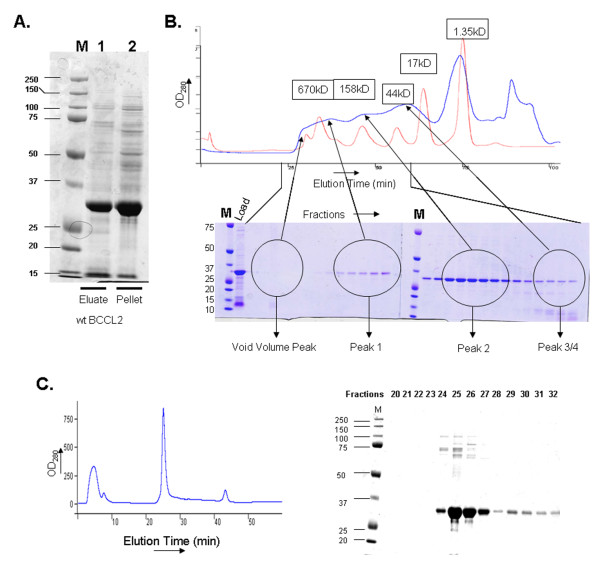
**Purification of the BCCL2 protein expressed in bacteria**. **A**. Bacterial cells expressing the BCCL2 protein were lysed and the homogenates subjected to purification approaches. In lane 1, the soluble BCCL2 protein was purified by Ni^+2^-NTA metal-affinity chromatography. Lane 2 shows the insoluble pellet obtained after lysis of the bacteria with lysis buffer. The proteins in each sample were resolved by SDS-PAGE and Coomassie Blue staining. **B**. The affinity-purified BCCL2 protein was loaded onto a gel-filtration column and eluted at a flow rate of 0.3 ml/min. The OD_280 _of the eluted protein is plotted (blue line). The profile of the globular protein standards (thyroglobulin (670 kD), bovine gamma-globulin (158 kD), chicken ovalbumin (44 kD), equine myoglobin (17 kD) and vitamin B12 (1.35 kD) is shown in red. Fractions from the gel-filtration column were separated on a 12% SDS-polyacrylamide gel, which was stained with Coomassie Blue. An aliquot of the BCCL2 protein sample loaded on the gel-filtration column was analyzed (Load), along with the molecular-weight markers (M). **C**. The affinity-purified BCCL2 protein was loaded onto a Hi-trap Q anion-exchange column and eluted at a flow rate of 0.5 ml/min (left panel). The fractions from the column were separated on a 12% SDS-polyacrylamide gel, which was stained with Coomassie Blue (right panel).

TRIM5-21R is a chimeric protein containing the RING domain of TRIM21 and the B-box 2, coiled coil, linker 2 and B30.2(SPRY) domain of TRIM5α_rh _[28,29]. TRIM5-21R, like many TRIM proteins [20,21], is prone to aggregation at high concentrations. To identify constructs that might be better behaved, we characterized TRIM5-21R fragments that were relatively resistant to trypsin and chymotrypsin digestion [28]. Proteins corresponding to these protease-resistant fragments were expressed in E. coli. Among the several constructs tested, the BCCL2 protein was soluble and able to be purified in adequate amounts following affinity purification (Table 1). The BCCL2 protein consists of an N-terminal His_6 _tag and a vector-derived spacer fused to the B-box 2 domain, the coiled-coil domain and the linker 2 (L2) region of TRIM5α_rh _(^83^EVKLSPEE...MEFRLTDA^296^); the predicted molecular weight of the BCCL2 protein is 29.026 kD. Although the BCCL2 protein lacks a B30.2(SPRY) domain, which is needed for HIV-1 capsid binding and virus restriction [7,19,28,29], it retains the other TRIM5α domains required for inhibition of HIV-1 infection. The BCCL2 protein with an N-terminal His_6 _tag was expressed in the BL21(DE3) strain of E. coli under the control of the T7 promoter. Different temperatures, times/duration of induction, and IPTG concentrations were studied to identify conditions under which BCCL2 protein expression was maximal, while maintaining adequate solubility. Optimal conditions involved treatment with 1 mM IPTG at an optical density of the culture of 0.8 and an overnight induction at 18°C. Although a significant portion (approximately 60-70%) of the BCCL2 protein remained in the insoluble pellet, soluble BCCL2 protein (approximately 0.1 mg/liter of E.coli) was obtained and was affinity purified using Ni-NTA beads and elution with 250 mM imidazole (Figure 1A).

**Table 1:** Expression and solubility of rhesus monkey TRIM5α variants in E. coli

Construct^a^	Soluble^b^	Inclusion Body^b^
Full-length TRIM5α	+	+
RBCC	-	+++++
RBCCL2	+/-	++
RING-BCCL2	-	+
BCC	-	+++
BCCL2	+++	++++

**^a^**The rhesus monkey TRIM5α variants are designated according to the domains included in the protein: R = RING domain; B = B-box 2 domain; CC = coiled-coil domain; L2 = linker 2 region. All of the TRIM5α constructs were expressed in BL21(DE3)pLysS E. coli using the Champion pET Gateway vector, which adds an N-terminal hexa-histidine tag and a spacer. The inserted fragment of the RBCCL2 protein corresponds to the TRIM5α_rh _sequences ^1^MASGILL...MEFRLTDA^296^. The inserted fragment of the RING-BCCL2 protein corresponds to the TRIM5α_rh _sequences ^15^CPICLE...MEFRLTDA^296^.

**^b^**The amount of protein found in the soluble and insoluble (inclusion body) fractions is indicated, with increased levels designated by increasing numbers of plus signs.

The affinity-purified BCCL2 protein was analyzed by gel-filtration chromatography (Figure 1B). The BCCL2 protein migrated in overlapping broad peaks at positions corresponding to molecular weights of approximately 50 - 400 kD. The BCCL2 protein was further purified by anion-exchange chromatography, where it eluted in a single peak (Figure 1C). The purified protein fractions (fractions 24 to 27) were pooled, concentrated and stored at 4°C (short-term) or at -20°C at concentrations less than 0.5 mg/ml (long-term).

### Effects of B-box 2 changes on BCCL2 expression and oligomerization

**Figure 2:**
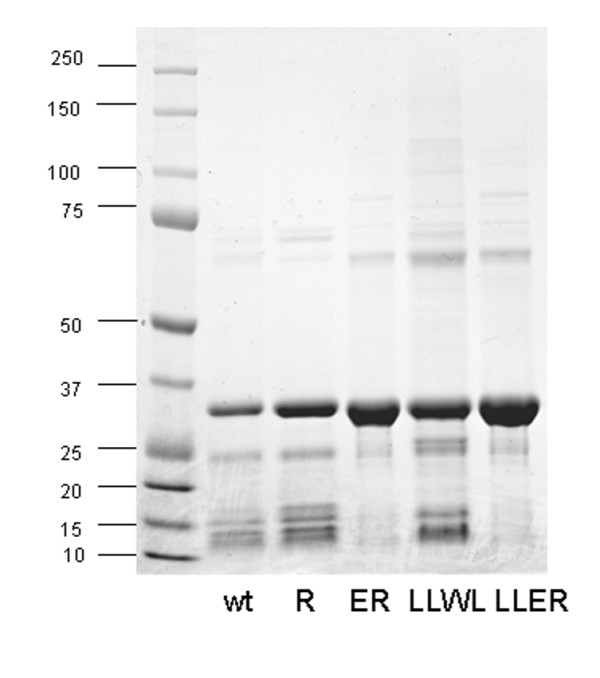
**Effect of B-box 2 changes on BCCL2 expression and solubility**. The wild-type (wt) BCCL2 protein and the indicated B-box 2 mutants were expressed in E. coli. The bacteria were lysed and the lysates centrifuged at 4000 × g for 10 minutes. The supernatants were loaded onto a Ni^+2^-NTA affinity column; the proteins eluted with 300 mM imidazole were analyzed by SDS-PAGE and Coomassie Blue staining.

**Figure 3:**
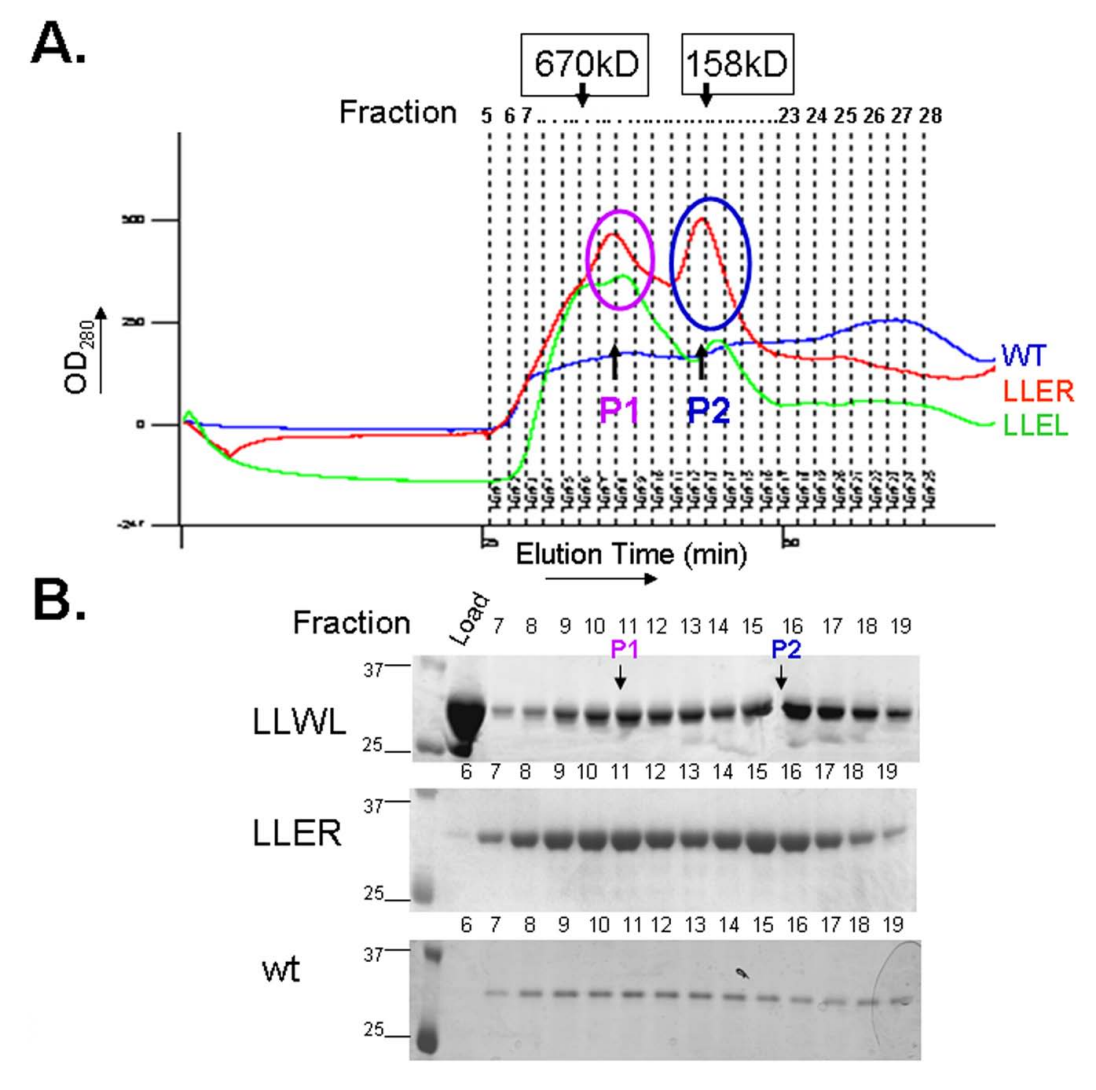
**Comparison of the size-exclusion chromatography profiles of the wild-type and mutant BCCL2 proteins**. **A**. Purified wild-type (wt) and mutant BCCL2 proteins were loaded onto a gel-filtration column and eluted at a flow rate of 0.3 ml/min. The protein peaks 1 (P1) and 2 (P2) are indicated. The positions at which the globular proteins standards thyroglobulin (670 kD) and bovine gamma-globulin (158 kD) were eluted in a parallel run are indicated. **B**. Fractions from the gel-filtration column were separated on a 12% SDS-polyacrylamide gel, which was stained with Coomassie Blue (bottom panel). The 25- and 37-kD molecular weight markers (M) are shown in the left-most lanes. An aliquot of the LLWL protein loaded on the gel-filtration column was also analyzed (Load). The positions of peaks P1 and P2 are noted.

The self-association of TRIM5α dimers in higher-order complexes is dependent upon specific charged and hydrophobic residues on the surface of the B-box 2 domain [16,26]. To investigate whether TRIM5α self-association might contribute to the behavior of the BCCL2 protein, we altered B-box 2 residues previously shown to contribute to higher-order association [16,26] (Table 2). Several-fold increases in the amount of the BCCL2 protein that could be solubilized resulted from the changes in the B-box 2 domain (Figure 2). The expression level of soluble B-box mutant proteins such as LLER was approximately 1-2 mg/liter of E.coli. The B-box 2 mutants (LLWL and LLER) with multiple surface changes eluted as two major peaks on a gel-filtration column, in contrast to the broad peak observed for the wild-type BCCL2 protein (Figure 3). The LLER protein demonstrated the clearest separation into two peaks of approximately 170 and 400 kD.

**Table 2:** B-box 2 variants of the BCCL2 protein

BCCL2 Variant	B-box 2 changes
wild-type (wt)	
ER	E120R, R121E
WL	W117E, L118S
WER	W117E, E120R, R121E
LLWL	L105E, L106E, W117E, L118S
LLER	L105E, L106E, E120R, R121E

### Effect of zinc on LLER expression and oligomerization

**Figure 4:**
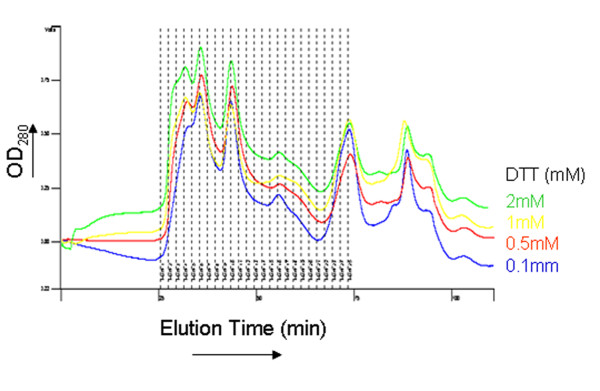
**Effect of zinc and dithiothreitol (DTT) on LLER oligomerization**. The LLER protein was applied to a gel-filtration column in the presence of 25 μM ZnCl_2 _and the indicated concentrations of DTT and eluted at a flow rate of 0.3 ml/min. The profile of the eluted protein is shown.

Because the B-box 2 domain of the BCCL2 protein binds two zinc ions [16], we investigated the effect of zinc supplementation and DTT treatment on the expression and oligomerization of the LLER variant. No augmentation of LLER expression resulted from supplementation of growth medium with 25-100 mM ZnCl_2_; in fact, LLER expression was diminished at the highest ZnCl_2 _concentration tested (data not shown). The addition of ZnCl_2 _or DTT did not significantly affect the elution profile of the LLER protein on gel-filtration chromatography (Figure 4 and data not shown).

### Dynamic light scattering analysis of the oligomeric state of the LLER protein

**Figure 5:**
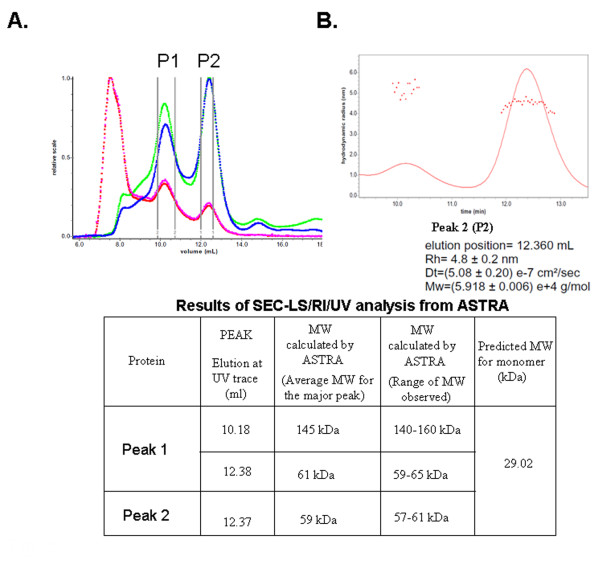
**Size-exclusion chromatography-light scattering (SEC-LS) analysis of the LLER protein**. **A**. Approximately 300 μg of the purified LLER protein was applied to a Superose 6 HR 10/30 column coupled with an in-line Dawn EOS laser light-scattering apparatus, refractometer and UV detector. The dashed red line represents the light-scattering signal at 90°. The green trace represents the refractive index. The blue trace represents the UV absorption at 280 nm. **B**. The solid red line indicates the refractometer trace of Peak 1 (P1) and Peak 2 (P2). The "dots" represent the weight-average molecular mass for each slice, measured every second. The results of the analysis performed on the protein in Peak 2 (P2) are shown beneath the figure. The table summarizes the analyses of Peaks 1 and 2.

The oligomeric state of the LLER protein, purified by nickel-affinity and anion-exchange chromatography, was investigated by size-exclusion chromatography-light scattering (SEC-LS). SEC-LS can estimate molecular mass independently of the Stokes radius of the protein and without reference to a curve based on protein standards. Molecular mass determination by SEC-LS depends only upon the light-scattering and refractive indices, which are measured by detectors situated downstream of the size-exclusion column. The LLER protein eluted in two peaks, the first ranging from 140-160 kD, the second from 57-61 kD (Figure 5A). A size analysis by Astra (Figure 5B) calculated a hydrodynamic radius of approximately 4.8 nm. The molecular weight of the protein in Peak 2 is consistent with that expected for a dimer, whereas the protein in Peak 1 apparently represents a higher-order oligomer.

**Figure 6:**
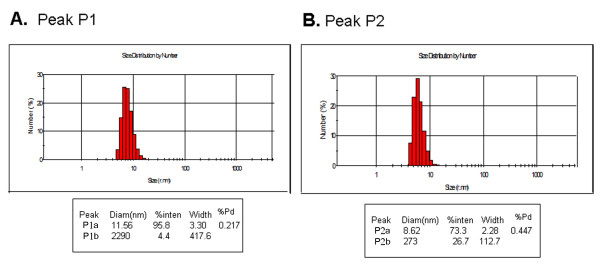
**Dynamic light scattering analysis of the LLER protein**. The polydispersity (%Pd) and size (mean and mode, in nm) of the purified LLER protein were estimated by dynamic light scattering. A 12-μl sample of a 500 μg/ml solution of the LLER protein in peak 1 **(A) **and peak 2 **(B) **were analyzed by a temperature-controlled Zetasizer Nano-S dynamic light-scattering instrument at 20°C.

We conducted additional dynamic light-scattering analysis of the two purified LLER protein fractions from size-exclusion chromatography (Figure 6). The protein in Peak 1 exhibited a diameter of 11.6 nm and a polydispersity of 22%, and the protein in Peak 2 exhibited a diameter of 8.6 nm and a polydispersity of 45%.

### Crosslinking analysis of the LLER protein

**Figure 7:**
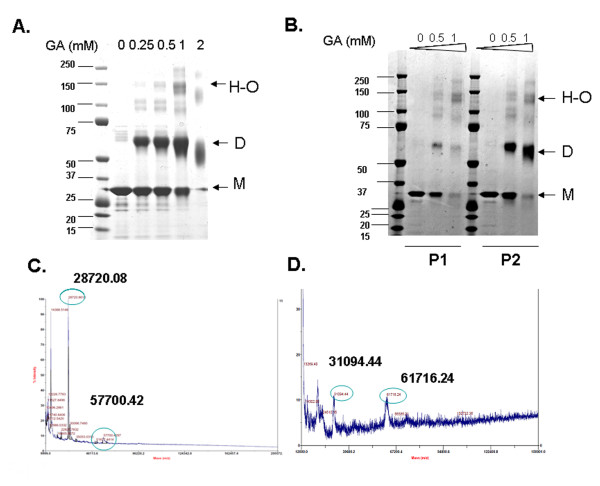
**Crosslinking analysis of the LLER protein**. **A**. The LLER protein, purified by nickel-affinity and anion-exchange chromatography, was incubated with the indicated concentrations (in mM) of glutaraldehyde (GA). The protein was then boiled in Laemmli buffer and analyzed on a 4-12% SDS-polyacrylamide gel. The arrow indicates the positions of the monomers (M), dimers (D) and higher-order oligomers (H-O). **B**. The peak 1 (P1) and peak 2 (P2) fractions of the LLER protein were purified by gel-filtration chromatography and separately crosslinked by incubation with the indicated concentrations of glutaraldehyde (GA). The proteins were then analyzed on a 4-12% SDS-polyacrylamide gel. **C, D**. The LLER protein was either untreated **(C) **or crosslinked with glutaraldehyde **(D) **prior to MALDI-TOF analysis. The molecular mass estimations in daltons for the single mass ionization peaks are shown.

The oligomeric state of the LLER protein was examined by chemical crosslinking with glutaraldehyde, followed by analysis on an SDS-polyacrylamide gel. The major crosslinked form of the protein migrated around 60 kD, suggestive of a dimer (Figure 7A). Smaller quantities of higher-order, approximately 150-kD forms were observed after crosslinking.

Next, the LLER protein from gel-filtration fractions (Peaks 1 and 2) were crosslinked with glutaraldehyde and analyzed. The LLER protein in Peak 2 mainly crosslinked into dimers. By contrast, the protein in Peak 1 formed fewer dimers and crosslinked primarily into higher-molecular-weight species (Figure 7B).

Because crosslinked TRIM5 protein can migrate aberrantly on SDS-polyacrylamide gels [27-29,68], the LLER protein crosslinked with glutaraldehyde was analyzed by mass spectrometry. Most of the untreated LLER protein exhibited a mass consistent with a monomer (28,720 Da), with a small amount of dimer (57,700 Da) (Figure 7C). The LLER protein crosslinked with glutaraldehyde demonstrated approximately equivalent amounts of monomer (31,094 Da) and dimer (61,716 Da) (Figure 7D). These results support the conclusion that the LLER protein is mainly dimeric, with some higher-order forms. Approximately half of the LLER protein migrated on native gels at a size consistent with a dimer, with most of the remainder of the protein migrating as a monomer; a small fraction of the protein migrated as a higher-order oligomer (data not shown).

**Figure 8:**
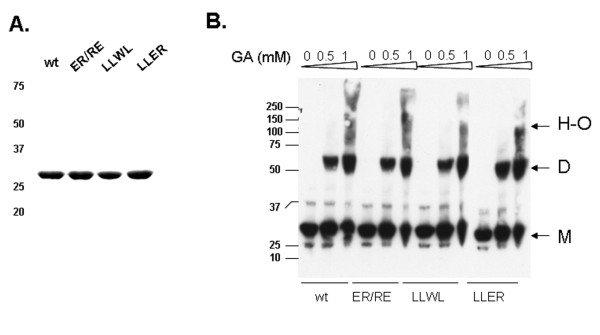
**Oligomerization state of BCCL2 variants in mammalian cells**. **A**. Lysates from 293 T cells expressing the wild-type (wt) and mutant BCCL2 proteins with V5 epitope tags were analyzed by 12% SDS-PAGE and Western blotted with an HRP-conjugated anti-V5 antibody. **B**. Lysates from 293 T cells expressing the wild-type (wt) and mutant BCCL2 proteins were treated with the indicated concentrations of glutaraldehyde and then boiled in Laemmli buffer and analyzed by SDS-PAGE and Western blotting, as described above. The positions of the molecular-weight markers in kD are shown. The arrows indicate the positions of monomers (M), dimers (D) and higher-order oligomers (H-O).

The oligomeric state of the wild-type BCCL2 protein was compared with that of the B-box 2 mutants. For this purposes, the proteins were expressed transiently in 293 FT human kidney cells. Compared with the wild-type BCCL2 protein, the B-box 2 mutants were expressed comparably (Figure 8A) and exhibited a similar pattern upon crosslinking (Figure 8B). Thus, all of the B-box 2 mutants retain the ability to dimerize.

### Circular dichroism (CD) Spectrometry

**Figure 9:**
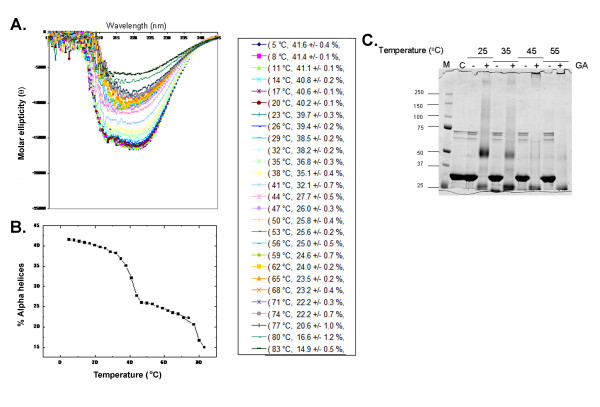
**Secondary structure and melting temperature of the LLER protein**. **A**. The far-UV spectra (195 - 245 nm) of the LLER protein were recorded with an Aviv circular dichroism (CD) spectrometer at the indicated temperatures. The percentage of alpha-helical content at the various temperatures was calculated and is shown in the key. **B**. The melting curve of the BCCL2 protein was generated by plotting the alpha-helical content as a function of temperature. Note the biphasic shape of the curve. **C**. Approximately 2 μg of the BCCL2 protein was incubated at the indicated temperatures for 5 minutes prior to the addition of 1 mM glutaraldehyde. Incubation at the same temperature was continued for another 8 minutes, after which the reaction was quenched by addition of excess 0.1 M Tris-HCl, pH 7.5 buffer. A control reaction without the addition of glutaraldehyde was also performed at each temperature. The reaction mixtures were boiled in Laemmli buffer and analyzed on a 4-12% SDS-polyacrylamide gel. M, molecular weight markers.

The CD spectrum of the LLER protein was determined at different temperatures (Figure 9A). The spectrum is characterized by a large negative change of ellipticity at 222 nm and is typical of that expected for a structured, predominantly alpha-helical, protein [69]. A quantitative analysis of the spectra obtained at different temperatures suggested that an extended alpha-helical conformation exists over a wide range of temperatures. The stability of the protein was examined by monitoring the effect of temperature on the far UV CD spectrum at 222 nm (Figure 9B). The LLER protein exhibited a biphasic loss of alpha-helical content, with one transition at 42°C and another near 80°C. The 42°C transition occurs concomitantly with a dramatic decrease in the dimerization of the protein, as judged by crosslinking analysis (Figure 9C).

### Protease resistance of the BCCL2 protein

**Figure 10:**
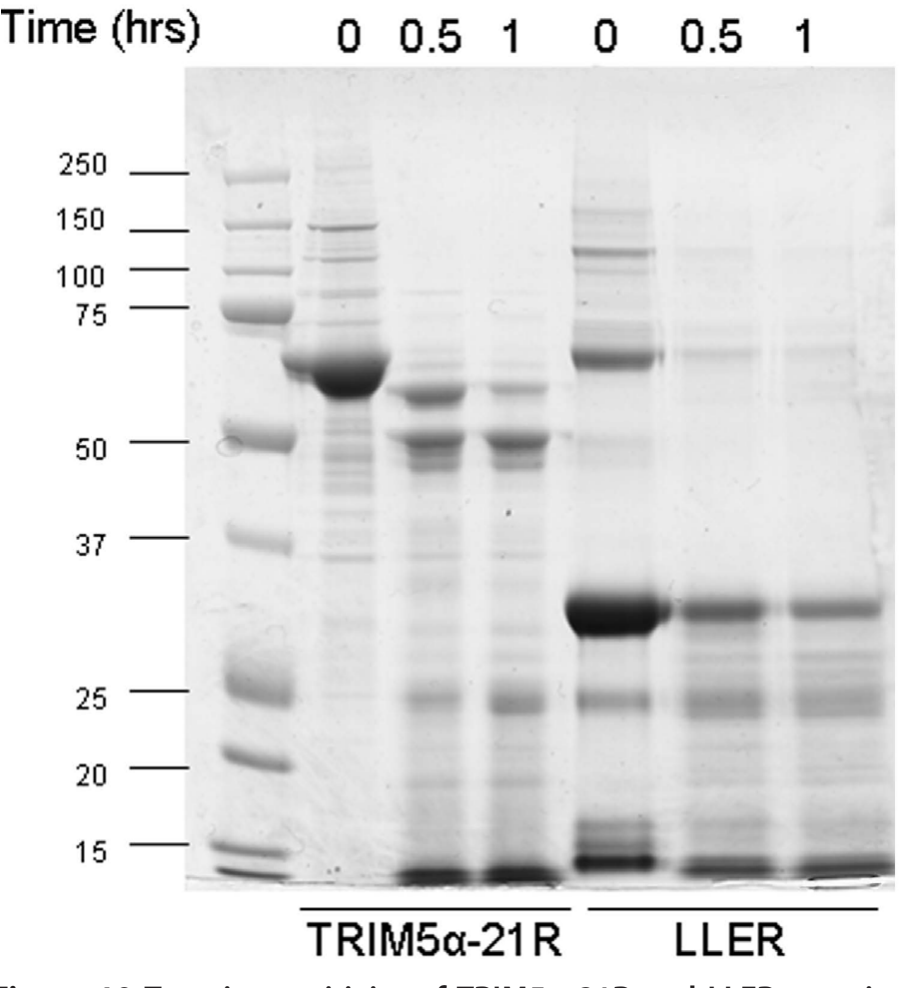
**Trypsin sensitivity of TRIM5α-21R and LLER proteins**. The TRIM5α-21R and LLER proteins were incubated at 37°C with a 1:150 (weight/weight) solution of trypsin in 25 mM bicarbonate buffer, pH 8 for the indicated times. The reaction was stopped by adding soybean trypsin inhibitor (STI) and the reaction mixtures were boiled in Laemmli buffer. The mixtures were then analyzed by SDS-PAGE and the gels were stained with Coomassie Blue.

The protease resistance of the LLER protein was compared with that of the TRIM5α-21R protein by incubating the proteins with trypsin. At incubation times where the TRIM5α-21R was substantially degraded to smaller fragments by trypsin, the LLER protein was retained in significant amounts (Figure 10). A major portion of the LLER protein is resistant to digestion, suggesting that these fractions are either compactly folded or represent higher-order oligomers that are resistant to trypsin digestion.

### Electron microscopy of the LLER protein

**Figure 11:**
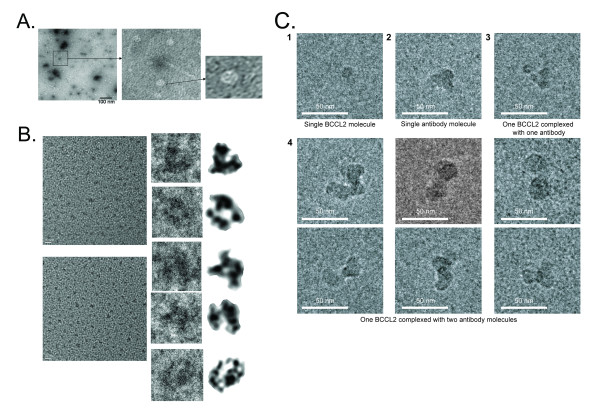
**Electron microscopy of the LLER protein**. **A**. The BCCL2 LLER protein purified by nickel-affinity, anion-exchange, and gel-filtration chromatography was applied to glow-discharged carbon grids. After staining with 1% uranyl formate, the grids were examined with a Tecnai G2 Spirit BioTWIN electron microscope (FEI Company) at 100 kV. **B**. The cryoelectron microscopic images of the LLER protein were taken at a magnification of 150,000 × and at a defocus of 3~5 μm with a Tecnai F20 field-emission gun electron microscope operating at 200 kV. The proteins that were purified as described above were embedded in a thin ice film on a Quantifoil grid, using an FEI Vitrobot, a robot that swiftly plunges the protein-loaded grid into liquid ethane. The images were low-pass filtered with background noises removed (right column). The bars in the left-hand images are 20 nm. **C**. The Peak 2 fraction of the LLER protein was incubated with an anti-His_6 _antibody and imaged by single-particle cryoelectron microscopy, as described above. Representative images of the LLER protein alone (panel 1), the antibody alone (panel 2), and the LLER protein complexed with one or two antibody molecules (panels 3 and 4, respectively) are shown.

The purified LLER protein was examined by negative staining under a transmission electron microscope. Most of the shapes on the grid were round or ovoid, although occasional filaments were observed (Figure 11A). Single-particle imaging by cryoelectron microscopy was performed on the peak 1 and peak 2 gel-filtration fractions of the LLER protein. The LLER proteins from peak 1 formed C-shaped structures roughly 120-130 Å wide, with an appendage asymmetrically placed to one side of the arc (Figure 11B). The LLER proteins in peak 2 consisted of spherical structures approximately 105-115 Å in diameter (Figure 11C, panel 1). The addition of antibodies directed against the N-terminal His_6 _tag allowed visualization of complexes of the LLER protein with one antibody molecule (Figure 11C, panel 3) or with two antibody molecules (Figure 11C, panel 4). These results indicate that these structures represented LLER multimers that were at least dimeric.

## Discussion

Previous studies of the TRIM5α-21R protein suggested that TRIM5α preferentially forms dimers [28,29]. One of the major protease-resistant fragments of TRIM5α-21R consisted of the B-box 2 and coiled-coil domains, as well as the adjacent linker 2 (L2) region [28]. Here, we produced this protease-resistant fragment (BCCL2) of rhesus monkey TRIM5α and verified that it is relatively resistant to trypsin digestion compared with the TRIM5α-21R protein. Protease resistance often is associated with the absence of conformationally flexible surface loops on a protein and thus might indicate a propensity to crystallize [70,71]. Although the polydispersity of the purified BCCL2 protein is high, an unfavorable predictor of crystallizability [71,72], additional modifications of the protein, optimization of solvent conditions or the inclusion of appropriate ligands may facilitate crystallization.

Our results demonstrate that, in the absence of other eukaryotic proteins, the BCCL2 protein forms dimers. Thus, the B-box 2 and coiled-coil domains, as well as the adjacent L2 region, are sufficient for TRIM5 dimerization. Neither of the terminal domains (i.e., RING or B30.2(SPRY)) is required for efficient dimerization. The estimates of the secondary structure content of the BCCL2 protein at different temperatures are consistent with predictions that the coiled-coil domain of TRIM proteins is mostly alpha helical [65-67]. Exposure to 45°C resulted in a dissociation of the dimer and a decrease in the helicity of the protein. The integrity of the dimer interface and that of a subset of the alpha-helical structures on TRIM5 may be interconnected. Such a situation might be expected if dimeric contacts exist along the exposed surface of the alpha helices of each subunit of the dimer.

The BCCL2 dimers formed higher-order structures. Recently it has been reported that the full-length rhesus monkey TRIM5α protein forms dimers, trimers, hexamers and multimers of higher complexity in mammalian cells; the hexameric form in particular appeared to be the most abundant multimer [30]. TRIM5α multimerization does not involve disulfide bonds and is not affected by infection with restriction-sensitive viruses [30]. The formation of the functional TRIM5 higher-order oligomers depends upon specific B-box 2 amino acid residues [16,26], several of which were altered in the BCCL2 protein without apparent disruption of the higher-order oligomerization. However, multiple changes in the B-box 2 surface did decrease the amount of large BCCL2 aggregates. This allowed more homogeneous preparations of a BCCL2 variant, LLER, to be analyzed by electron microscopy. Single-particle analysis revealed that the lower-molecular-weight form of LLER consisted of approximately 10-nm spheres. These LLER forms likely represent dimers; consistent with this, many examples of complexes consisting of two anti-His_6 _antibodies bound to a single LLER sphere were evident. The TRIM5 coiled coil, which is approximately 100 amino acid residues in length, could not be accommodated in these 10-nm spheres as a linear, rod-like structure; the existence of insertions at specific locations in the coiled coil regions of TRIM5 variants is consistent with the possibility that the coiled coil bends or folds back upon itself. The higher molecular weight LLER complexes formed C-shaped structures, with an appendage protruding from the convex side of the arc. The size of these structures is larger than that expected for a dimer, and possibly represents a pentamer or hexamer. We do not know if these higher-order structures are biologically relevant; it may be that the TRIM5α domains (RING and B30.2(SPRY)) not included in the BCCL2 construct influence higher-order self-association either quantitatively or qualitatively. Future work will explore these issues.

## Conclusions

The central core of TRIM5α, consisting of the B-box 2, coiled coil and L2 linker region, forms dimers that are largely alpha helical. The melting of the alpha-helical structure of this TRIM5α core was biphasic, with one transition at 42°C associated with a loss of dimerization, and a second transition at 80°C.

## Abbreviations

TRIM: tripartite motif; HIV: human immunodeficiency virus; SIV: simian immunodeficiency virus; SEC: size-exclusion chromatography; BCCL2: B-box 2 - coiled coil - linker 2; HRP: horseradish peroxidase; CD: circular dichroism; MALDI-TOF: matrix-assisted laser desorption ionization-time of flight; PBS: phosphate-buffered saline.

## Authors' contributions

AK, YM, GB, LW and JS designed the experiments. AK, YM and GB performed the experiments. All of the authors participated in the interpretation of the data. AK, LW, and JS wrote the manuscript. All authors read and approved the final manuscript.
